# An Electronic Tongue Designed to Detect Ammonium Nitrate in Aqueous Solutions

**DOI:** 10.3390/s131014064

**Published:** 2013-10-18

**Authors:** Inmaculada Campos, Lluis Pascual, Juan Soto, Luis Gil-Sánchez, Ramón Martínez-Máñez

**Affiliations:** 1 Centro de Reconocimiento Molecular y Desarrollo Tecnológico (IDM), Unidad Mixta Universidad Politécnica de Valencia–Universidad de Valencia de Valéncia, Camino de Vera s/n, E-46022 Valencia, Spain; E-Mails: incasan2@upvnet.upv.es (I.C); llpasvi@upvnet.upv.es (L.P.); jsotoca@upv.es (J.S.); lgil@eln.upv.es (L.G.-S.); 2 CIBER de Bioingeniería, Biomateriales y Nano medicina (CIBER-BBN), Bellaterra, E-08193 Barcelona, Spain; 3 Departamento de Química. Universidad Politécnica de Valencia. Camino de Vera s/n., E-46022 Valencia, Spain; 4 Departamento de Ingeniería Electrónica. Universidad Politécnica de Valencia, Camino de Vera, s/n. E-46022 Valencia, Spain

**Keywords:** ammonium nitrate, electronic tongue, PCA, fuzzy ARTMAP, PLS

## Abstract

An electronic tongue has been developed to monitor the presence of ammonium nitrate in water. It is based on pulse voltammetry and consists of an array of eight working electrodes (Au; Pt; Rh; Ir; Cu; Co; Ag and Ni) encapsulated in a stainless steel cylinder. In a first step the electrochemical response of the different electrodes was studied in the presence of ammonium nitrate in water in order to further design the wave form used in the voltammetric tongue. The response of the electronic tongue was then tested in the presence of a set of 15 common inorganic salts; *i.e.*; NH_4_NO_3_; MgSO_4_; NH_4_Cl; NaCl; Na_2_CO_3_; (NH_4_)_2_SO_4_; MgCl_2_; Na_3_PO_4_; K_2_SO_4_; K_2_CO_3_; CaCl_2_; NaH_2_PO_4_; KCl; NaNO_3_; K_2_HPO_4_. A PCA plot showed a fairly good discrimination between ammonium nitrate and the remaining salts studied. In addition Fuzzy Art map analyses determined that the best classification was obtained using the Pt; Co; Cu and Ni electrodes. Moreover; PLS regression allowed the creation of a model to correlate the voltammetric response of the electrodes with concentrations of ammonium nitrate in the presence of potential interferents such as ammonium chloride and sodium nitrate.

## 1. Introduction

Ammonium nitrate (NH_4_NO_3_) is generally found in the form of odourless, transparent, hygroscopic deliquescent crystals or white granules. NH_4_NO_3_ is the cheapest source of oxygen available in a condensed form for commercial explosives. The original experiments with NH_4_NO_3_, as a component of explosive mixtures began in the second half of the nineteenth century. Grindel and Robin were the first to use NH_4_NO_3_ in explosive formulations as a replacement of potassium nitrate in black powder [1] and in 1867, the Sweedish chemists Ohlosson and Norrbin patented an explosive called *Ammoniakus* [2]. The explosive properties of NH_4_NO_3_, such as sensitivity to detonation, impact and heat, rate of detonation, *etc.* were reported first in the beginning of the last century. NH_4_NO_3_ is usually used by itself, in conjunction with fuels or in mixtures with solid fuels and sensitizers such as nitroglycerine and TNT [3]. The use of NH_4_NO_3_ with fuel oil provides a very widely used explosive usually called ANFO. Aluminum is also sometimes added to increase the sensitiveness of the explosive mixture. Though the idea of using NH_4_NO_3_ with a fuel as a commercial explosive formulation was proposed as early as 1867 (*vide ante*), ANFO explosives were only finally developed in 1955. They can be made on-site in a mobile unit and are inexpensive and safe to handle. However, they have low strength and detonation velocity and cannot be used in the presence of water [4,5]. The detonation velocity was improved by the creation of water-based explosives. These explosives were developed by mixing NH_4_NO_3_ solutions with oils in different compositions. The micro-droplets of the emulsion explosive offer the advantage of intimate contact between fuel and oxidizer and outperform conventional water based slurries. In fact, ANFO and water-based commercial explosives have largely displaced the nitroglycerine-based dynamites, thanks to their low cost, safety, versatile performance and application.

Identification and quantification of explosives has constituted an emerging and important topic of interest due to their relevant role in security threat. Moreover, it has been widely discussed that the detection of explosive compounds is a highly significant task in forensics and antiterrorist activities. As the danger of terrorism is increasing, the demand for reliable and rapid methods for screening luggage or to analyze suspect samples is also growing up. Apart of the classical procedures for the individual detection of nitrate and ammonium, several methods have been reported in the literature for the simultaneous speciation of NO_3_^−^ and NH_4_^+^, among them, perhaps the most common in the use of ion chromatography [6]. In general ion chromatography method offers good reproducibility, high sensitivity and selectivity [7,8]. Nevertheless, for the determination of these inorganic nitrogen species two separate sets must be applied: one with an anion-exchange column for the determination of NO_3_^−^ and another with a cation-exchange column for the determination of NH_4_^+^. Hence, the simultaneous determination of nitrogen species requires a sophisticated assembly. Moreover these methods use reagents and expensive columns that must be periodically cleaned and calibrated as their performances change with time. These technical requirements unfortunately did not permit a routine and *in situ* or *at site* use of these procedures [9–11].

Electronic tongues (ETs) are multisensory systems, which consist of a number of low-selectivity sensors and use advanced mathematical procedures for signal processing based on Pattern Recognition and Multivariate data analysis. These electronic devices are inspired by the human taste system and as in nature ET, are constituted of a limited number of receptors that can respond to several stimuli of different nature. The first voltammetric electronic tongue was proposed by Winquist and used metallic electrodes to detect different samples of several juices, milk and phosphate buffer [12].

Following these concepts and taking into account our interest in the design of sensing systems and probes for the detection of explosives [13–15] and our experience in the design of electronic tongue devices for several applications [16–18], we report herein the use of an electronic tongue for the discrimination of NH_4_NO_3_ *versus* other common salts in an aqueous environment. The aim of this work is develop a rapid, simple and low-cost method for the detection of levels of ammonium nitrate in water samples using voltammetry as analytical tool. The electronic tongue is based in voltammetry and uses a set of simple metallic electrodes (Au, Pt, Rh, Ir, Cu, Co, Ag and Ni). Multivariate analysis including Cross validation, Principal Components Analysis and Partial Least Square (PLS) techniques have also been applied in this study.

## 2. Experimental Section

### 2.1. Sample Preparation

Discrimination studies were carried out using 15 inorganic salts, *i.e.*, NH_4_NO_3_, MgSO_4_, NH_4_Cl, NaCl, Na_2_CO_3_, (NH_4_)_2_SO_4_, MgCl_2_, Na_3_PO_4_, K_2_SO_4_, K_2_CO_3_, CaCl_2_, NaH_2_PO_4_, KCl, NaNO_3_, K_2_HPO_4_ (with purity of 99.9% from Sigma-Aldrich, Madrid, Spain). All these samples were prepared at a concentration of 10^−2^ mol·L^−1^ in water. The samples were measured in quintuplicate.

Moreover quantification studies were carried out via a factorial experimental design approach which was applied to create a system of three compounds/three levels. By using the experimental design (MODDE 8.0, Umetrics, Umeå, Sweden) the number of samples was generated. This design takes into account the possible interactions between the analytes and provides the minimum number of solutions which represent the system and determine the concentration of each compound in each sample. The final set included 20 samples prepared by addition of three selected compounds (NH_4_NO_3_, NaCl and NH_4_Cl) into phosphate buffer (Na_2_HPO_4_, 10^−2^ mol·L^−1^ at pH 7). The Table 1 displays the concentration levels of the samples which were: blank, low (1 × 10^−3^ mol·L^−1^), medium (5 × 10^−3^ mol·L^−1^) and high (1 × 10^−2^ mol·L^−1^). All samples were measured once and in random order to minimize possible error due to memory of the electrodes.

### 2.2. Electronic Tongue Based on Pulse Voltammetry

**Figure 1.**
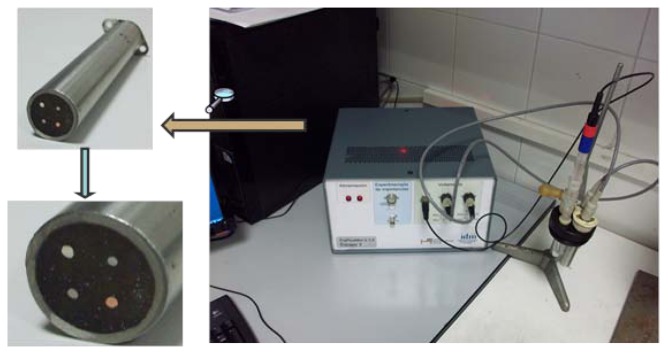
The electronic tongue formed by non-noble electrodes.

The electronic tongue device used in this work consists of eight working electrodes array (Au, Pt, Rh, Ir, Cu, Co, Ag and Ni with purity of 99.9% and 1 mm diameter from Sigma-Aldrich) housed inside a stainless steel cylinder used at the same time as both the body of the electronic tongue system and as counter-electrode (See Figure 1). The electronic tongue was inspired in a similar design reported by Winquist *et al.* [12]. Noble electrodes (*i.e.*, Au, Pt Rh and Ir) are selected due to their capability to adsorb different chemical species on their surface. Moreover non-noble electrodes (Cu, Co, Ag and Ni) were also used due to the potential occurrence of chemical reactions between species in the solution and the oxidised metal. In addition a Saturated Calomel Electrode (SCE) was used as reference electrode.

The generation of pulses and recording of current data were performed in a potentiostat-galvanostat Autolab (Eco Chemie, Utrecht, The Netherlands), controlled with the General Purpose Electrochemical System software (GPES Version 4.9). The measuring procedure was carried out by applying 10 pulses of fixed potential to the electrodes (range of pulses from −0.5 to +0.4 V relative to the SCE electrode, with duration of 50 ms). A total of 4800 currents (60 points for pulse × 10 pulses × 8 electrodes) were recorded for each sample. The dimensions of the data sets were 40 × 4,800 and 20 × 4,800 for discriminant and quantification studies, respectively. Whereas the dimensions of the final data set (including only the four electrodes which provides better results) were 40 × 2,400 and 20 × 2,400, respectively.

Before use, the electrode surface was prepared by mechanical polishing with an emery paper, and rinsed with distilled water. Then it was polished on a felt pad with 0.05 μm alumina polish from BASi (West Lafayette, IN, USA), washed with distilled water and polished again on a nylon pad with 15, 3 and 1 μm diamond polishes, to produce a smooth, mirror-like electrode surface. Later in the development of series of measurements, only a simple diamond polishing was made.

### 2.3. Cyclic Voltammetry Studies

The electrochemical characterization of NH_4_NO_3_ solutions were performed using an Autolab PGSTAT100 instrument (Eco Chemie, Utrecht, The Netherlands). The electrochemical characterization was carried out using aqueous solution 0.01 mol·L^−1^ of ammonium nitrate at pH 7 buffered with phosphate at a concentration of 0.01 mol·L^−1^. All measurements were performed at room temperature (20 ± 2 °C). The electrochemical experiments were carried out using the electronic tongue (containing a set of electrodes housed into a homemade stainless steel cylinder). A SCE was used as reference electrode. The cyclic voltammetry experiments were carried out at a scan rate of 100 mV·s^−1^.

### 2.4. Data Management

Multivariate data analysis (MVDA) was used to treat the raw data obtained from the instruments. Principal component analysis (PCA) is an example of such an MVDA which explains the variance in the experimental data [19]. The PCA produces a score plot that is visualizing differences between the observations or experiments. This can be used for classifications or groupings of the observations. The first principal component (PC1) is the dimension along which the observations are maximally separated, or spread out. The second principal component (PC2) is the linear combination with maximal variance in a direction orthogonal to the first principal component, and so on [20].

For the instrumental data, the used data matrix was consisted of the number of experiments as number of objects, and the obtained current responses as variables [21]. Data were standardized since standardization gives all the variables the same variance and all the variables have the same influence of the estimation of the components.

A widely used method for the classification of different samples is artificial neural networks. This kind of networks is formed by mathematical algorithms which coefficients relate the values of multiple inputs with different output categories. To work with neural networks a training stage is required which sets the value of these coefficients, and a subsequent validation step which shows the network response to new data.

There are different types of networks, such as Fuzzy-Artmap type. This type of network is based on the so-called adaptive resonance theory (ART) [22], which develops models capable of rapid stable learning of recognition categories in response to arbitrary sequences of input patterns. The Fuzzy Artmap combines two ART units into a supervised learning structure where the first unit takes the input data and the second unit takes the correct output data [23] and it has demonstrated to show a good behaviour with low computational cost for a small number of data and groups of varying size [24].

To analyze the power of classification of the neural network applied to the data measured ‘leave-one-out’ cross-validation technique was applied. This cross-validation method extracts one data vector and the training of the network is done with the remaining data, in our case data are 74 measurements. The validation of the network is done with the extracted data. The validation is to determine if the network classifies correctly the extracted data. This process is repeated by using all the data, in our case 75 times. The discrimination capacity of the network is determined by the percentage of accurate classifications of data.

For quantitative analysis studies PLS techniques were applied. The main objective of PLS regression is to predict Y from X, by simultaneous decomposition of those matrixes or vectors in a group of components (latent variables) which explain as much as possible the covariance of X and Y [25]. Prediction models are created by using the calibration set (standards) and the collected data. Prior to build the model, “leave-one-out” cross validation technique was used to evaluate the adequacy of the experimental data, and to select the quantity of latent variable, indicating that 7 latent variables explain the 89% of the accumulated variance. All statistical analysis was performed with the software Solo (version 6.5, Eigenvector Research, Inc., Wenatchee, WA, USA).

## 3. Results and Discussion

### 3.1. Voltammetric Studies

In order to explore more in detail the response of the metallic electrodes in the presence of NH_4_NO_3_, the electrochemical behaviour of this salt was studied in water by cyclic voltammetry (10^−2^ mol·L^−1^ phosphate buffer at pH 7) using Au, Pt, Rh, Ir, Cu, Co, Ag and Ni as working electrodes. As it is well-known, the voltammetric response of a certain compound depends on the intrinsic chemical nature of the both the electrode and the electrochemical characteristics of the redox-active species. The electronic tongue device relies in the concept that subtle differences between relatively similar compounds might be reflected in the specific differential voltammetric response of the electrode ensemble.

**Figure 2.**
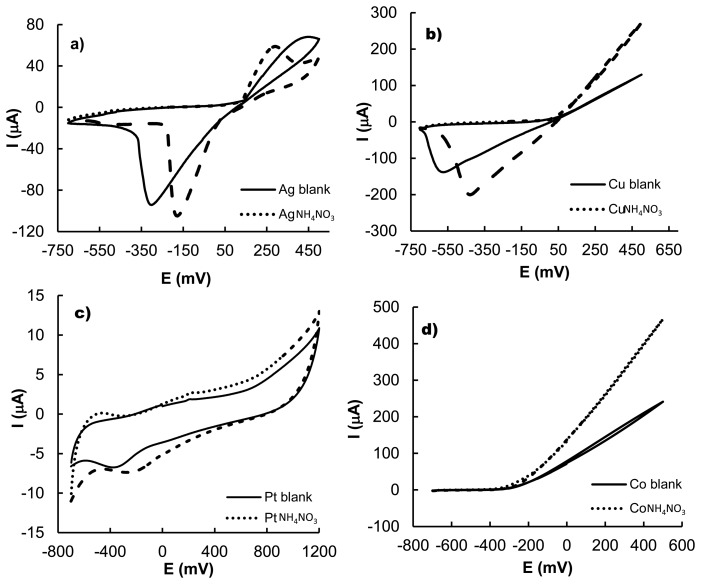

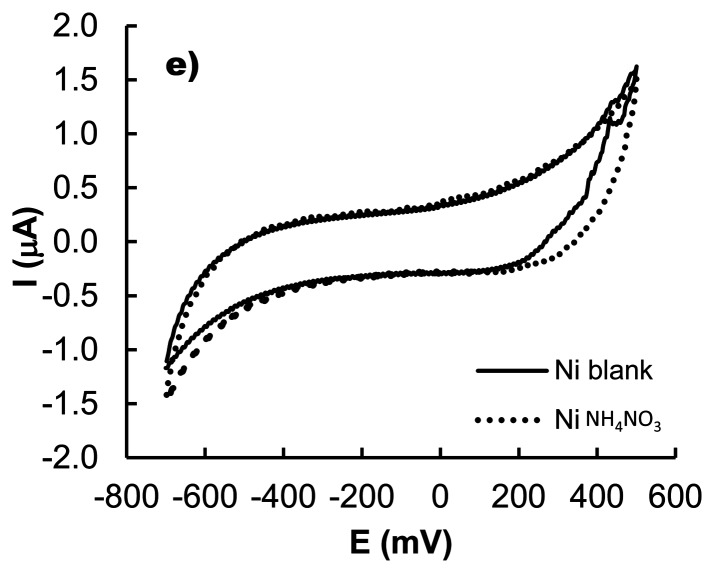
Cyclic voltammograms of the solvent (pH 7 buffered with 0.01 mol·L^−1^ phosphate buffer (solid line) and of 0.001 mol·L^−1^ NH_4_NO_3_ solutions (dashed line) measured at 100 mV·s^−1^ using silver (**a**), copper (**b**), platinum (**c**), cobalt (**d**) and nickel (**e**).

In the cyclic voltammetry studies, ammonium nitrate displayed an oxidation peak at 250 mV and a reduction process at −160 mV *vs.* SCE using Ag as electrode. This behaviour was clearly different to that found when Cu was used. In the latter case a reduction peak at −450 mV *vs.* SCE was found together with an increase in the corrosion of the copper electrode (see Figure 2). When a Co electrode was used, the presence of ammonium nitrate also caused an increment in the corrosion of the metal which started at −200 mV but in this case no reduction peaks were found. For platinum as electrode two small redox peaks were found at −250 and −500 mV. Finally, when nickel electrode is used as working electrode, the ammonium nitrate does not show the presence of redox peaks and only changes in the ohmic current was observed. Voltammetry studies on NH_4_NO_3_ with other electrodes tested (not shown) displayed minor changes in the ohmic current. Nevertheless it has to be taken into account that in recent studies it has been pointed out that the lack of clear redox processes does not hamper the use of voltammetric electronic tongues bearing in mind that changes induced by chemical species, in the currents from the reduction or oxidation of water, in the equilibrium potential of the electrodes or the presence of non-Faradic effects due to chemical adsorption on the electrode, might also be used for multivariate data analysis and chemical discrimination [15]. The performed electrochemical studies suggested that the use of these metals, in an electronic tongue format, may be suitable to detect ammonium nitrate in solution and to discriminate its presence from the existence of other salts.

### 3.2. Waveform Design

**Figure 3.**
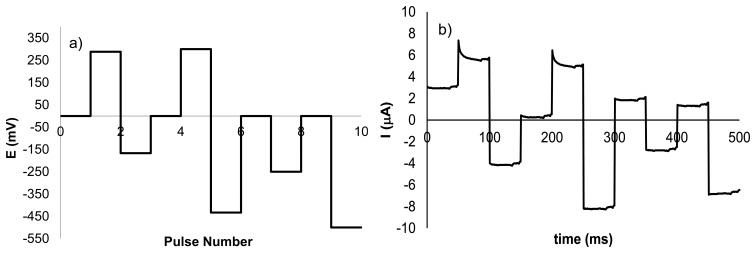
(**a**) The applied potentials, and (**b**) the current response of waveform applied to a sample that contains NH_4_NO_3_ (C = 1 × 10^−2^ mol·L^−1^) when a Ni electrode is used.

In previous works, we have demonstrated the importance of applying a suitable set of pulses in the electronic tongue in order to be able to detect certain compound in solution [16]. With this concept in mind, the waveform used in the discrimination of NH_4_NO_3_ was designed using the electrochemical response found for NH_4_NO_3_ in the voltammetric studies described above. Figure 3a shows the sequence of pulses used in the electrochemical tongue. Pulses 2 and 3, with values of 288 and −167 mV, respectively, were selected bearing in mind the corresponding oxidation and reduction peaks when a silver electrode was used. Pulses 5 and 6, having a potential of 300 and −433 mV were related with the redox processes observed for NH_4_NO_3_ with the copper electrode. Ammonium nitrate provided a small peak at −250 mV (pulse 8) when platinum electrode was used. The pulse of −500 mV was introduced due to the presence of a small increment in current in the presence of NH_4_NO_3_ observed for the Pt electrode. Finally, pulses with potential of 0 mV were added in other to obtain information about non-faradic processes. Figure 3b shows the current intensity when the waveform is applied to a sample with concentration 1 × 10^−2^ mol·L^−1^ using as working electrode a Ni electrode.

### 3.3. Spontaneous Clustering with PCA

In order to show the differential response of the tongue towards these different salts, the voltammetric electrochemical response was combined to form ensembles for pattern recognition in an attempt to find selectivity fingerprints using PCA analysis.

**Figure 4.**
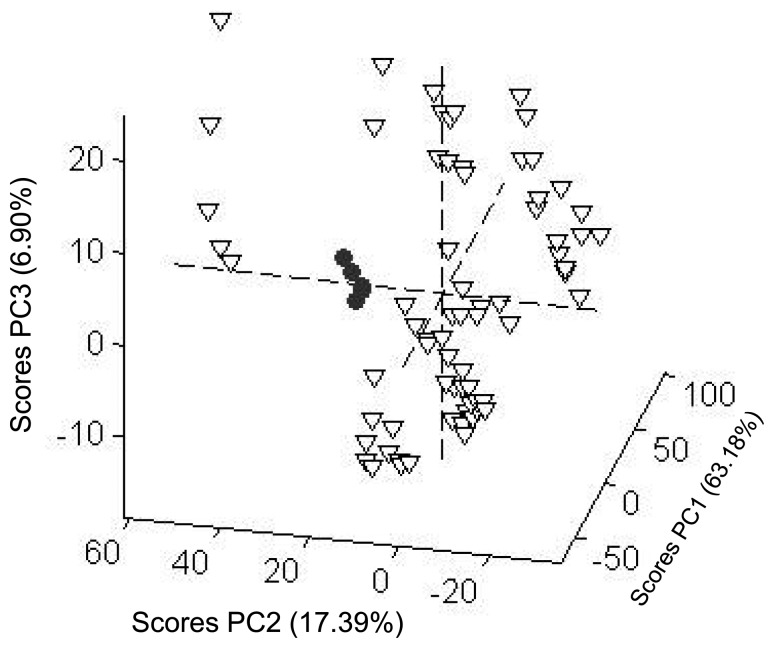
3 PCA score plot of NH_4_NO_3_ (dots) and the remaining 14 salts (triangles) using all the electrodes.

The PCA score plot of the results obtained from the voltammetric tongue in the presence of the selected salts (vide ante) is shown in Figure 4 for five different trials. The first principal component contained only 63.18% of the variance and the first three components represented 87.47% of total variance of the data. The three dimensions PC1, PC2 and PC3 in PCA plot were enough to differentiate the NH_4_NO_3_ samples. From Figure 4 it can be observed that the samples corresponding to the ammonium nitrate salt (represented by dots) appeared well separated, whereas the remaining salts (triangles) are distributed in different sites in the PCA plot. This is a promising result that suggests that the differential response in the voltammetric technique, using a set of electrodes, might be a suitable method for the detection of NH_4_NO_3_ in aqueous samples.

### 3.4. Fuzzy Artmap Analysis

**Figure 5.**
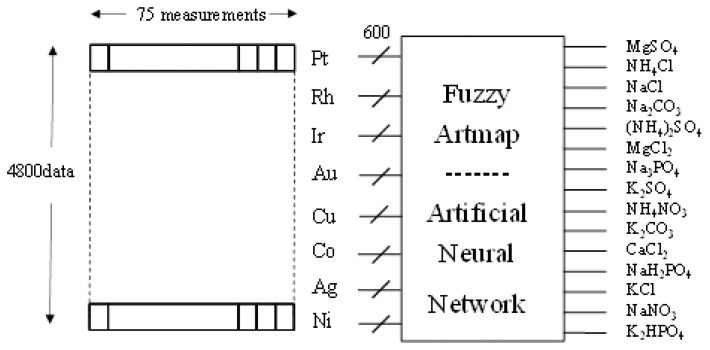
Classification of measures by Artificial Neural Networks accord the fifteen salts.

In addition to PCA, a quantitative analysis of the electronic tongue discriminatory power was carried out by means of artificial neural networks. For this purpose a neural network was developed to analyze the data obtained from the electronic tongue to determine which type of salt corresponds of the fifteen samples of the different salts analyzed. The neuronal network type used was Fuzzy-Artmap. The network uses a training step with a set of measurements and a further step for validation. For each measurement, a vector of 4,800 data points enters the network and it tries to determine to which type of solution corresponds through 15 digital outputs. This process is repeated with the rest of the measurements, so that the activated output will have the value of “one” and the not activated outputs will have “zero” value (See Figure 5).

The network was implemented in-house using function macros from basic functions of Matlab [25,26]. Table 1 displays the correct classification of each analyzed salt (outputs) calculated using (i) the whole set of eight electrodes; (ii) using only the noble metals electrodes (Pt, Rh, Ir and Au) and (iii) the non-noble metals electrodes (Cu, Co, Ag and Ni). Table 1 also shows the classification obtained when a combination of 4 selected electrodes (Pt, Cu, Co and Ni) was used. This latter selection was carried out taking into account the relatively good classification that was observed when using this specific combination of electrodes.

Table 1 shows how the different samples (five samples) of the different salt are correctly classified using the leave-one-out cross-validation technique. In general it was observed that non-noble metals displayed a better classification than when only noble metals were used. Besides, the better classification was observed when all the electrodes or the combination of Pt, Cu, Co and Ni electrodes were employed. In that latter case a total of 59 samples (74.6% success ratio bearing in mind all salts) were correctly classified. Moreover in terms of NH_4_NO_3_ classification the best results were found when using the signals from the Pt, Cu, Co and Ni metals (in this case all five samples were correctly classified) whereas the use of other combination of electrodes provided worse results. The good classification performance when using Pt, Cu, Co and Ni electrodes is most likely due to the selected potential pulses. The applied waveform was designed form the electrochemical information of cyclic voltammetry where these electrodes provide clear redox responses.

To further test the classification power of the electronic tongue, NH_4_NO_3_ samples (five samples) were attempted to be classified when they were compared with the five samples of a certain salt. Following this approach 14 tests were performed by comparison between NH_4_NO_3_ samples with the remaining 14 salts resulting in a total of 140 classifications. The analysis was performed separately using all electrodes and only the response obtained from the Pt, Cu, Co and Ni electrodes. The result of this analysis is shown in Table 2. When all the salts and all the electrodes were taken into account a total of 117 from 140 samples were correctly classified (83.57%), whereas when only the set of Pt, Cu, Co and Ni metals were considered 132 from 140 samples (94.28%) were correctly classified. These results point towards a fairly good classification of the NH_4_NO_3_ solutions when compared individually with solutions of other salts.

### 3.5. PLS Analysis

Encouraged by the obtained results in terms of classification of solutions containing the NH_4_NO_3_ explosive, we further completed our study by using partial least squares (PLS) techniques in an attempt to establish a correlation between the voltammetric measurements and the concentration of NH_4_NO_3_ in aqueous solution. Besides, this study was carried out using different mixtures of NH_4_NO_3_, NaCl and NH_4_Cl. With this objective in mind, 20 standard solutions were prepared by addition of the three selected compounds (NH_4_NO_3_, NaCl and NH_4_Cl) into phosphate buffer 10^−2^ mol·L^−1^ at three different levels; *i.e.*, blank, low (1 × 10^−3^ mol·L^−1^), medium (5 × 10^−3^ mol·L^−1^) and high (1 × 10^−2^ mol·L^−1^). The content of each salt and its concentration was designed by using the experimental design program MODDE 8.0. PLS prediction models were created from the experimental data collected from the response in solution of the Pt, Cu, Co and Ni electrodes.

For a PLS calibration the information from the concentration values is introduced into the calculation via the so-called latent variables. The number of latent variables in PLS is a parameter of the procedure whose value is to be estimated from the calibration data. According to cross-validate variance studies, 7 latent variables have been used for create the models.

**Figure 6.**
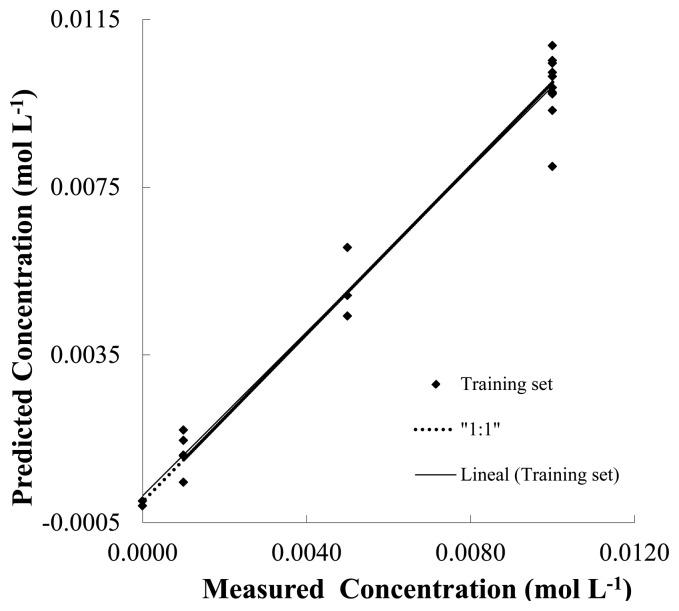
Plot score of prediction model of NH_4_NO_3_ (predicted *versus* real concentration) and linear fitting in water (pH 7, phosphate buffer 10^−2^ mol·L^−1^) employing the response observed from the Pt, Cu, Co and Ni electrodes using a set of different mixtures containing NH_4_NO_3_, NaCl and NH_4_Cl salts.

The Figure 6 shows the plot score of prediction model of NH_4_NO_3_ (predicted *versus* real concentration) and linear fitting in water (pH 7, phosphate buffer 10^−2^ mol·L^−1^) using the response observed from the Pt, Cu, Co and Ni electrodes using a set of different mixtures containing NH_4_NO_3_, NaCl and NH_4_Cl salts. Besides, Table 3 shows the results of prediction for the salts NH_4_NO_3_, NaCl and NH_4_Cl in terms of linear fitting parameters. In this context, a simple way to analyze the PLS prediction is to use a linear model (*i.e.*, y = p1x + p2) in order to adjust the predicted *vs.* the real concentration data shown in Figure 6.

In this model p1 is the slope of the curve fitting and p2 the intercept. In PLS analysis, when closer to 1 are the slopes and closer to 0 are the intercept, more accurate is the calibration model. Additionally the following figures also show the correlation coefficient (r^2^) and the root mean square error of cross-validation (RMESCV), which represents the goodness of the fit. These last parameters are a useful tool for evaluating the precision of predictions since it represents the error in the confidentiality of the model. A simple visual inspection of the spread of the experimental points along the straight line (reference line) in Figure 6 shows an accurate prediction in the concentration of NH_4_NO_3_. As shown Table 4, this precise prediction can be confirmed by the relatively low values of the RMSECV and the *p1*, slope of the curve, coefficients that are close to 1. Table 3 also shows good fitting parameters from the PLS analysis using Pt, Co, Cu and Ni electrodes for the prediction of concentrations of NaCl and NH_4_Cl salts.

## 4. Conclusions

A protocol to detect and quantify of NH_4_NO_3_ in aqueous samples is proposed here. The method consists of the use of a voltammetric electronic tongue based in a set of simple metallic electrodes. PCA analysis showed the capability of the electronic tongue to detect the presence of NH_4_NO_3_ in water and discriminate this compound from other common inorganic salts, *i.e.*, MgSO_4_, NH_4_Cl, NaCl, Na_2_CO_3_, (NH_4_)_2_SO_4_, MgCl_2_, Na_3_PO_4_, KSO_4_, NH_4_NO_3_, K_2_CO_3_, CaCl_2_, NaH_2_PO_4_, KCl, NaNO_3_, K_2_HPO_4_. In addition to PCA studies, a quantitative analysis of the electronic tongue discrimination ability was carried out by means of artificial neural networks using the leave-one-out cross-validation technique. A good discrimination of NH_4_NO_3_ against other salts when using the electrochemical response of a combination of four selected electrodes (Pt, Cu, Co and Ni) was found. Finally PLS data analysis using three compounds (NH_4_NO_3_, NH_4_Cl and NaCl) at three concentration levels showed a fairly good accurate concentration prediction for NH_4_NO_3_ in water samples. These results suggested that voltammetric electronic tongues could be useful for the detection of NH_4_NO_3_ in aqueous environments and pointed towards the possibility that this, or similar tongue-based systems, could be of application for the detection of NH_4_NO_3_ in real explosive formulation samples.

## Figures and Tables

**Table 1.** Concentrations of samples prepared by addition of three selected compounds (NH_4_NO_3_, NaCl and NH_4_Cl) into water (phosphate buffer 10^−2^ mol·L^−1^, pH 7).

**Sample**	**NH_4_NO_3_(mol·L^−1^) × 10^−3^**	**NaCl (mol·L^−1^) × 10^−3^**	**NH_4_Cl (mol·L^−1^) × 10^−3^**
1	10	10	1
2	1	10	10
3	5	5	5
4	10	1	5
5	1	10	1
6	10	5	10
7	10	10	10
8	10	10	10
9	10	10	10
10	1	1	1
11	1	1	10
12	10	1	10
13	10	10	10
14	1	1	5
15	5	1	10
16	5	1	1
17	10	1	1
18	10	1	10
19	0	0	0
20	0	0	0

**Table 2.** Number of successful in the discrimination of several salts using different combination of electrodes.

**Sample**	**All Electrodes**	**Noble Metals**	**Non-Noble Metals**	**Pt, Cu, Co and Ni**
MgSO_4_	3	0	4	5
NH_4_Cl	5	3	4	5
NaCl	3	2	3	2
Na_2_CO_3_	3	3	4	5
(NH_4_)_2_SO_4_	5	3	4	5
MgCl_2_	5	5	4	5
Na_3_PO**_4_**	5	5	5	5
K_2_SO**_4_**	2	0	3	0
**NH_4_NO_3_**	**4**	**1**	**4**	**5**
K_2_CO**_3_**	5	3	3	4
CaCl_2_	4	3	4	4
NaH_2_PO_4_	5	5	3	5
KCl	2	2	3	2
NaNO**_3_**	4	2	3	2
K_2_HPO**_4_**	4	3	3	5

Total	59(78.6%)	40(53.3%)	54(72%)	59 (78.6%)

**Table 3.** Number of successful classification in the discrimination of NH_4_NO_3_ when compared with different salts.

**Samples**	**All Electrodes**	**Pt, Cu, Co and Ni Electrodes**
MgSO_4_	9	9
NH_4_Cl	7	9
NaCl	9	9
Na_2_CO_3_	10	10
(NH_4_)_2_SO**_4_**	7	9
MgCl_2_	7	10
Na_3_PO**_4_**	10	10
K_2_SO**_4_**	9	9
K_2_CO**_3_**	9	10
CaCl_2_	9	10
NaH_2_PO_4_	9	10
KCl	8	8
NaNO**_3_**	8	9
K**_2_**HPO**_4_**	9	10

Total	117/140 (83.57%)	132/140 (94.28%)

**Table 4.** Parameters from the linear fitting of prediction data from the PLS analysis using Pt, Co, Cu and Ni electrodes.

	**r^2^**	**Slope (p1)**	**Intercept (p2) × 10^−4^(M)**	**RMSECV × 10^−3^(M)**
NH_4_NO_3_	0.977	0.9768	1.3	6.45
NaCl	0.987	0.9873	1.9	4.8
NH_4_Cl	0.984	0.9842	0.9	5.4
